# Perioperative blood loss of surgical techniques designed for alignment that do not violate intramedullary cavity of the femur in patients with total knee arthroplasty: A systematic review and meta-analysis

**DOI:** 10.1097/MD.0000000000042722

**Published:** 2025-08-01

**Authors:** Sang Gyu Kwak, Jae Bum Kwon, Hee Chan Kim Seo, Won-Kee Choi

**Affiliations:** a Department of Medical Statistics, College of Medicine, Daegu Catholic University, Daegu, Korea; b Department of Orthopaedic Surgery, College of Medicine, Daegu Catholic University, Daegu, Korea; c Department of Orthopaedic Surgery, Armed Forces Daegu Hospital, Daegu, Korea.

**Keywords:** bleeding, hemoglobin, meta-analysis, total knee arthroplasty, transfusion

## Abstract

**Background:**

We aimed to conduct a systematic review and meta-analysis comparing perioperative blood loss between navigation or robot-assisted total knee arthroplasty (TKA), or patient-specific instrumentation (PSI), which are surgical techniques that do not violate the femoral medullary cavity, and conventional TKA.

**Methods:**

The PICO (population, intervention, comparison, and outcome) of this study are as follows. (1) Population: patients undergoing primary unilateral TKA. (2) Intervention: navigation or robot-assisted TKA or PSI that do not violate intramedullary cavity of the femur. (3) Comparison: traditional TKA surgical techniques that violate intramedullary cavity of the femur. (4) Outcome: perioperative blood loss or hemoglobin reduction or transfusion rate during admission.

**Results:**

In the 16 studies, 640 participants who underwent surgery not violating intramedullary cavity of the femur (navigation or robot-assisted TKA or PSI) and 637 participants who underwent surgery violating intramedullary cavity of the femur. There was statistically significant difference in perioperative blood loss. The effect size of the perioperative blood loss for all cases by 2 groups was −150.65 (95% CI: −219.53 to −81.77, *P*-value < .001). The effect size of the perioperative blood loss on the second day after surgery by 2 groups was −164.29 (95% CI: −320.62 to −7.96, *P*-value = .040). There was statistically significant difference in hemoglobin between the 2 groups before surgery and 1 day after surgery. The effect size of the difference in hemoglobin between before surgery and 1 day after surgery by 2 groups was −0.20 (95% CI: −0.38 to −0.02, *P*-value = .030).

**Conclusions:**

Navigation or robot-assisted TKA, or PSI, exhibited lower perioperative blood loss compared to conventional TKA. Additionally, they showed lower decreases in hemoglobin levels postoperatively. With these findings, it can be concluded that navigation or robot-assisted TKA, or PSI, may be considered as a selective option for reducing postoperative bleeding in TKA.

## 1. Introduction

The amount of blood loss following total knee arthroplasty (TKA) varies widely, as well as the frequency of homologous blood transfusion.^[1]^ It is known that receiving homologous blood transfusion after TKA is associated with increased postoperative complications and length of hospital stay.^[2]^ For this reason, surgeons are making various efforts to reduce perioperative bleeding during TKA.^[3,4]^ The surgical technique performed by the surgeon is also known as a significant factor determining the amount of perioperative bleeding.^[5]^ Many studies have reported that performing TKA using navigation or robot-assisted techniques, or patient-specific instrumentation (PSI), reduces perioperative bleeding compared to conventional TKA.^[6–8]^ Navigation or robot-assisted TKA, as well as PSI, are developed to achieve optimal femorotibial alignment.^[9]^ With this surgical approach during TKA, there is theoretical advantage of reduce bleeding within the femoral cavity as there is no need for an intramedullary guiding rod.^[10]^ However, many clinical studies show disagreement regarding this point.^[11,12]^ In light of this, the authors aim to conduct a systematic review and meta-analysis comparing perioperative blood loss between navigation or robot-assisted TKA, or PSI, which are surgical techniques that do not violate the femoral medullary cavity, and conventional TKA.

The authors aim to investigate the following points.

1. Determine if navigation or robot-assisted TKA and PSI result in less perioperative blood loss compared to conventional TKA.
2. Investigate whether navigation or robot-assisted TKA and PSI result in less short-term decrease in hemoglobin levels after surgery compared to conventional TKA.
3. Determine if there is a lower chance of blood transfusion during the hospitalization period for surgery in TKA using navigation or robot-assisted techniques and PSI compared to conventional TKA.

Since this meta-analysis is not a clinical study involving human subjects, a protocol for IRB approval was not needed.

## 2. Materials and methods

### 2.1. Registration and reporting guidelines

The authors wrote the protocol to carry out the meta-analysis and registered the protocol on the international prospective register of systematic reviews site (https://www.crd.york.ac.uk/PROSPERO/) (ID: CRD42024535184). Also, transparent reporting of systemic review and meta-analysis (PRISMA Checklist: https://www.prisma-statement.org/) was completed and attached as a supplementary file.

### 2.2. Search strategy

The PICO (population, intervention, comparison, and outcome) of this study are as follows.

1. Population: patients undergoing primary unilateral TKA.
2. Intervention: navigation or robot-assisted TKA or PSI that do not violate intramedullary cavity of the femur.
3. Comparison: traditional TKA surgical techniques that violate intramedullary cavity of the femur.
4. Outcome: perioperative blood loss or hemoglobin reduction or transfusion rate during admission.

Articles published between January 1, 1990, and March 30, 2024 were searched in PubMed, Cochrane, and Embase using the following key phrases (Table 1).

**Table 1:** Articles were searched in PubMed using the following key phrases.

Pubmed; search on March 30, 2024	
Search	Query
#1	“Total knee arthroplasty” [Ti/Ab] or “Total knee replacement” [Ti/Ab]
#2	“blood loss” [Ti/Ab] or “bleeding” [Ti/Ab] or “transfusion” [Ti/Ab]
#3	“robot” [Ti/Ab] or “robotic” [Ti/Ab] or “navigation” [Ti/Ab] or “patient specific instrumentation” [Ti/Ab] or “computer assisted surgery” [Ti/Ab]
#4	#1 and #2 and #3

### 2.3. Inclusion and exclusion criteria

The following studies were included in this study:

1. Studies involving patients undergoing primary unilateral TKA with techniques designed for alignment that do not violate intramedullary cavity of the (Robot, Navigation, PSI).
2. Studies comparing the intervention group (Robot, Navigation, PSI) with a control group (primary TKA with conventional surgical techniques that using intramedullary guiding rod).
3. Studies of blood loss, decrease in hemoglobin levels, and blood transfusion after TKA.
4. Studied of randomized controlled trials.

The exclusion criteria were as follows:

1. Review articles, case reports, protocols, and conference presentations.
2. Studies published in languages other than English.

### 2.4. Data extraction

Data for meta-analysis were independently investigated by 2 researchers (W.K.C. and S.G.K.). Duplicate studies were excluded, and studies that met the eligibility criteria were selected. Studies were evaluated for eligibility by reviewing the title and abstract. After reading the full text, studies were finally selected for inclusion in the meta-analysis, and discrepancies were resolved through discussion. Research characteristics, study design, number of patients (intervention and control groups), schedules of acupuncture treatment, outcome measurements, time and number of measurements were investigated.

Outcome variables were the quantitative variables of intraoperative blood loss (mL), perioperative blood loss (mL), hemoglobin (g/dL), and length of hospital stay (days), and the qualitative variables of blood transfusion (Y/N). In the papers, when the values for intraoperative blood loss (mL) and perioperative blood loss (mL) were expressed in L units, they were multiplied by 1000 and unified into mL units.

When the median (m), minimum value (a), and maximum value (b) were presented for a quantitative variable, the mean and standard deviation (SD) values were calculated using the following formula.^[13]^


$$\begin{array}{l} Mean=\frac{a+2m+b}{4}\;\;\;and\;\;\;SD=\sqrt{\frac{1}{12}\left(\frac{{\left(a-2m+b\right)}^{2}}{4}+{\left(b-a\right)}^{2}\right)}. \end{array}$$

When the mean and the upper bound and lower bound values of 95% confidence interval were presented, the standard deviation (SD) value was calculated using the following formula. Here, $z_{\alpha /2}$ is the value of the point corresponding to α/2 on the standard normal distribution.


$$\begin{array}{l} SD=\frac{\left(Upper\;\;\;bound-mean\right)}{z_{\alpha /2}}\times \sqrt{n}. \end{array}$$

When the range (*r*) using the minimum and maximum values was presented, the mean and standard deviation (SD) values were calculated using the following formula.^[14]^ Here f is the tabulated conversion factor.


$$\begin{array}{l} SD=f\times r. \end{array}$$

We collected the values of the hemoglobin (g/dL) presented in the paper according to the evaluation time points. Then, the changes from preoperation to postoperative day 1, day 2 and days 3 to 5 were calculated. Since this corresponds to the amount of change in the dependent group, the mean value and standard deviation for change between preoperation and postoperation were calculated using the following formulas and the correlation was derived from different study which presented the standard deviation for change.


$$\begin{array}{l} Mean_{change}=Mean_{pre-operation}-Mean_{post-operation}. \end{array}$$

$$\begin{array}{lll} & SD_{change}\; & =\sqrt{SD_{pre-operation}^{2}+SD_{post-operation}^{2}-2\times Correlation\times SD_{re-operation}\times SD_{post-operation}}. \end{array}$$

### 2.5. Quality assessment

The methodological quality was evaluated using revised Cochrane risk of bias tool (ROB 2.0). The potential sources of bias are listed in the followings; bias arising from the randomization process (D1), bias due to deviations from intended intervention (D2), bias due to missing outcome data (D3), bias in measurement of the outcome (D4), and bias in selection of the reported result (D5). The judgement of bias was described as one of the followings: “low risk of bias,” “some concerns,” “high risk of bias,” or “No information.”^[15]^

### 2.6. Statistical analysis

The Review Manager (RevMan v.5.3 software) was used for statistical analysis. Heterogeneity test was performed using $I^{2}$ statistics for each analysis, measuring the degree of inconsistency. If the value of $I^{2}$ statistics was close to 100%, it was considered as having substantial heterogeneity, and the random-effects model was applied for data analysis. If the value of $I^{2}$ statistics was close to 0%, pooled data was considered as homogenous, and the fixed effects model was used for data analysis. We analyzed the mean difference of perioperative blood loss, intraoperative blood loss, length of hospital stays, blood transfusion and hemoglobin. Further, the 95% confidence interval (CI) was used in the analysis. For the evaluation of publication bias, the funnel plot and the egger test were used. *P*-value < .05 was considered statistically significant.

Root mean square error (RMSE) was derived from the effect size and total effect size for sensitivity analysis b. $Total\;\;\;effect\;\;\;size_{\left(i\right)}\;\;\;$ was calculated by excluding the i-th study, i = 1,..., k (k = total number of studies). The following formula was used for calculation of RMSE.^[16]^


$$\begin{array}{l} RMSE=\sqrt{\frac{1}{k}\underset{i=1}{\overset{k}{\sum }}\;{\left(Total\;\;\;effect\;\;\;size-Total\;\;\;effect\;\;\;size_{\left(i\right)}\right)}^{2}} \end{array}$$

If the RMSE value close to 0 indicates that the individual study has little effect on the total effect size. In other words, it means that there is no variability due to inclusion and exclusion of the total effect size in 1 study, it means that it is robust and insensitive. Conversely, higher RMSE values indicate greater variability in the total effect size, which means that the total effect size is not robust and can vary widely between individual studies.

## 3. Results

**Figure 1.**
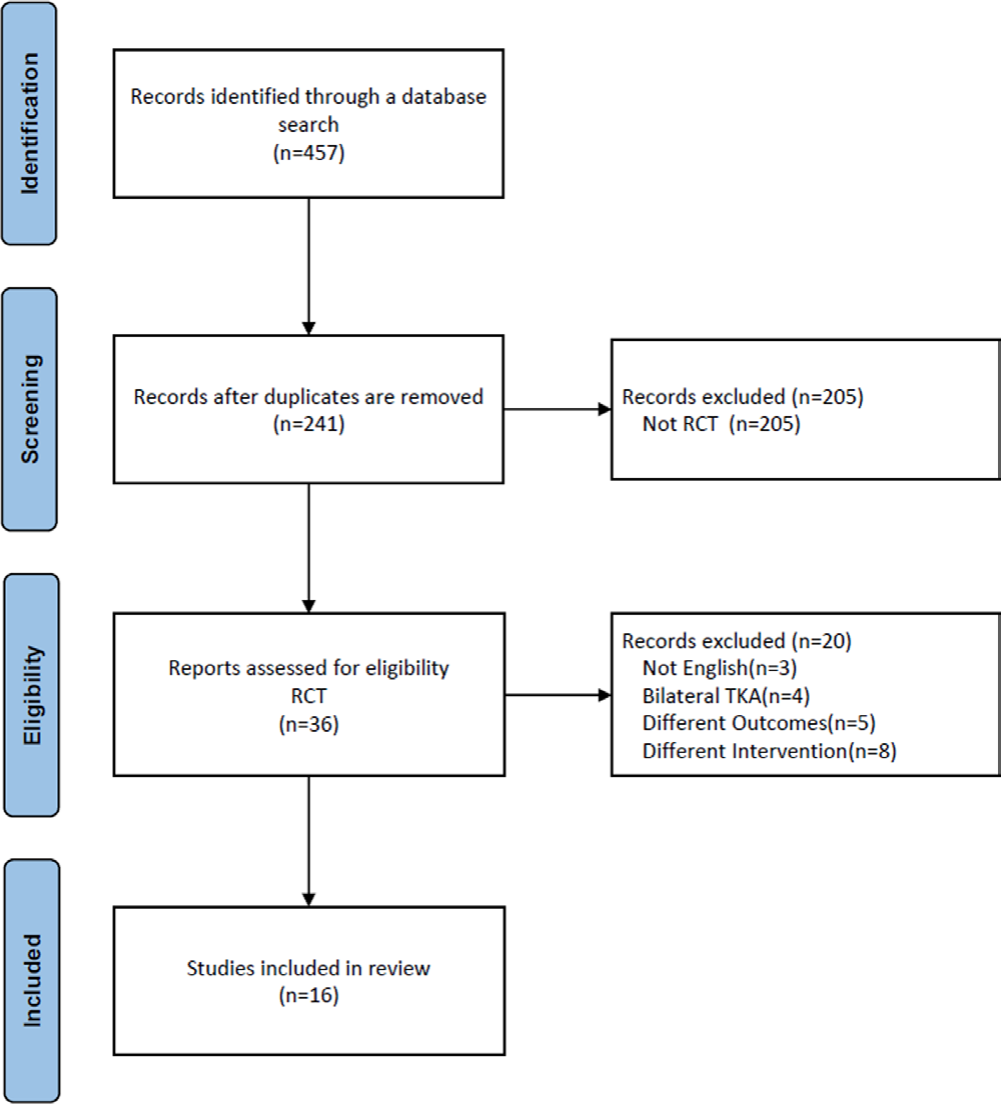
PRISMA diagram for the systematic review and meta-analysis. RCT = randomized controlled trial.

In the databases, 457 articles were searched, and 216 duplicated articles were removed (Fig. 1). After screening for eligibility based on a review of the title and abstract, 36 articles of randomized controlled trials were included for full-text reading. After a detailed assessment, 20 articles were excluded. Accordingly, 16 studies were finally included in our meta-analysis. The characteristics of the studies included in the research also described in Table 2.^[11,17–31]^

**Table 2:** Characteristics of included RCTs.

Reference	Intervention	Number of patients (intervention/control)	Outcome measurements	Time of Hgb measurements	Time of perioperative blood loss measurements
Yen et al^[17]^	Navigation	50/50	Perioperative blood loss Reduction of Hgb Post OP transfusion rate Length of hospital stay	POD1, POD2	Unknown
Xu et al^[18]^	RAT	37/35	Intraoperative blood loss Length of hospital stay		
Giannotti et al^[19]^	PSI	20/20	Perioperative blood loss		Unknown
Thiengwittayaporn et al^[20]^	Navigation	40/40	Perioperative blood loss		Unknown
Kotela et al^[21]^	PSI	49/46	Perioperative blood loss Post OP transfusion rate Length of hospital stay		POD 2
Singla et al^[22]^	Navigation	29/28	Perioperative blood loss Post OP transfusion rate Reduction of Hgb Intraoperative blood loss	POD2	Unknown
Kalairajah et al^[23]^	Navigation	30/30	Perioperative blood loss Reduction of Hgb	POD2	POD 2
Ferrara et al^[24]^	PSI	15/15	Perioperative blood loss Post OP transfusion rate Reduction of Hgb Intraoperative blood loss	POD1	POD 2
Ikawa et al^[25]^	Navigation	121/120	Perioperative blood loss		POD 7
Vide et al^[26]^	PSI	47/48	Reduction of Hgb Length of hospital stay	POD1	
Yuan et al^[27]^	RAT	28/32	Intraoperative blood loss		
Mitsiou et al^[11]^	Navigation	40/40	Perioperative blood loss Reduction of Hgb	POD1, POD2, POD5	POD 2
Conteduca et al^[28]^	Navigation	50/50	Perioperative blood loss		POD 2
Randelli et al ^[29^	PSI	31/29	Perioperative blood loss Reduction of Hgb	POD1, POD2, POD3	Unknown
Cucchi et al^[30]^	PSI	10/10	Perioperative blood loss Reduction of Hgb	POD1, POD2, POD3	POD 1
Hinarejos et al^[31]^	Navigation	43/44	Perioperative blood loss Reduction of Hgb	POD 4	POD 1

Hgb = hemoglobin, OP = operation, POD = postoperative day, PSI = patient-specific instrumentation, RAT = robotic assisted total knee arthroplasty, RCT = randomized controlled trial, TKA = total knee arthroplasty.

### 3.1. Study characteristics

As a result of reviewing title and abstracts, a total of 16 RCT studies were included. In the 16 studies, 640 participants who underwent surgery not violating intramedullary cavity of the femur (navigation or robot-assisted TKA or PSI) and 637 participants who underwent surgery violating intramedullary cavity of the femur. In 6 studies, the difference in hemoglobin was measured before surgery and on the first day after surgery.^[11,17,24,26,29,30]^ In 6 studies, the difference in hemoglobin was measured before surgery and on the second day after surgery.^[11,17,22,23,29,30]^ In 4 studies, the difference in hemoglobin was measured before surgery and 3 to 5 days after surgery.^[11,29–31]^ Four papers presented length of hospital stay. Thirteen papers measured and presented perioperative blood loss values, of which 2 studies presented perioperative blood loss values on the 1st day after surgery, 5 studies presented perioperative blood loss values on the 2nd day after surgery,^[11,21,23,24,28]^ 1 study presented perioperative blood loss values on the 7th day after surgery and 5 studies did not present measurement time. Intraoperative blood loss and blood transfusion were presented as results in 4 papers.

### 3.2. Quality assessment

**Figure 2.**
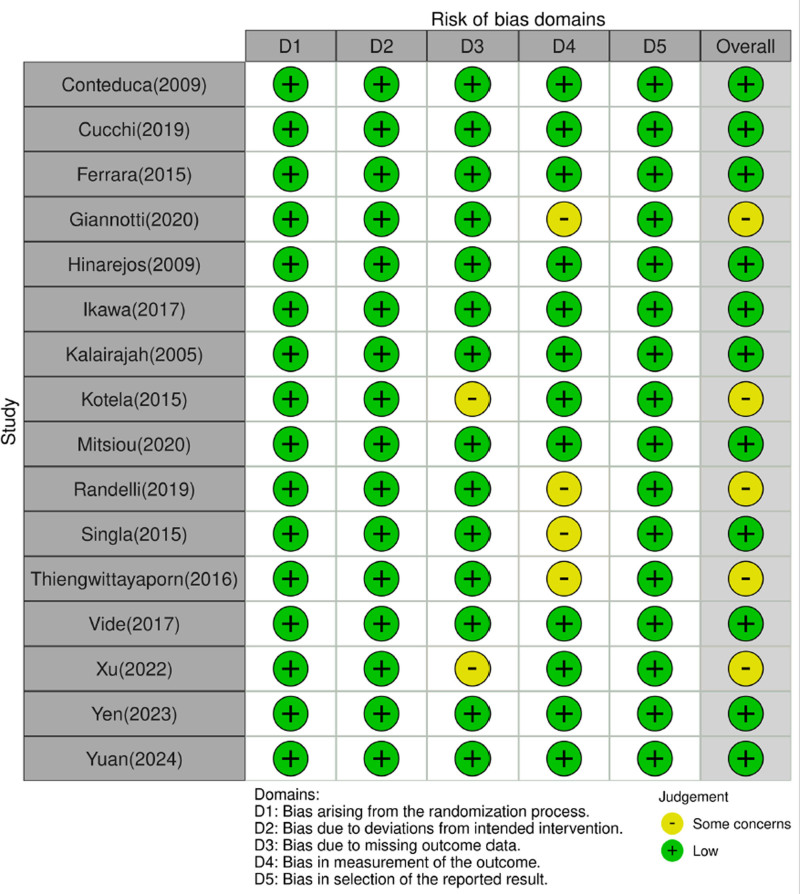
ROB traffic light plot depicting individual study findings.

The included studies were evaluated for 5 domains (D1–D5) using ROB 2.0 and robvis (risk of bias visualization tool: https://www.riskofbias.info/welcome/robvis-visualization-tool) (Fig. 2). Since all papers are randomized controlled trials, 3 out of 5 categories are “bias arising from the randomization process (D1),” “bias due to deviations from intended intervention (D2),” and “bias in selection of the reported result (D5)” was evaluated as low risk of bias. In 2 studies, the category “bias due to missing outcome data (D3)” was evaluated as some concern because there was no technology for how to deal with missing values even though there were missing values at the time of flow up in the results. In 4 studies, the “bias in measurement of the outcome (D4)” category was evaluated as having some concerns because there was no description of the exact measurement time for perioperative blood loss in the results. Therefore, the overall evaluation result among the 15 papers is that 10 papers were evaluated as “low risk of bias,” and 5 papers were evaluated as “some concerns.”

### 3.3. Meta-analysis results

**Figure 3.**
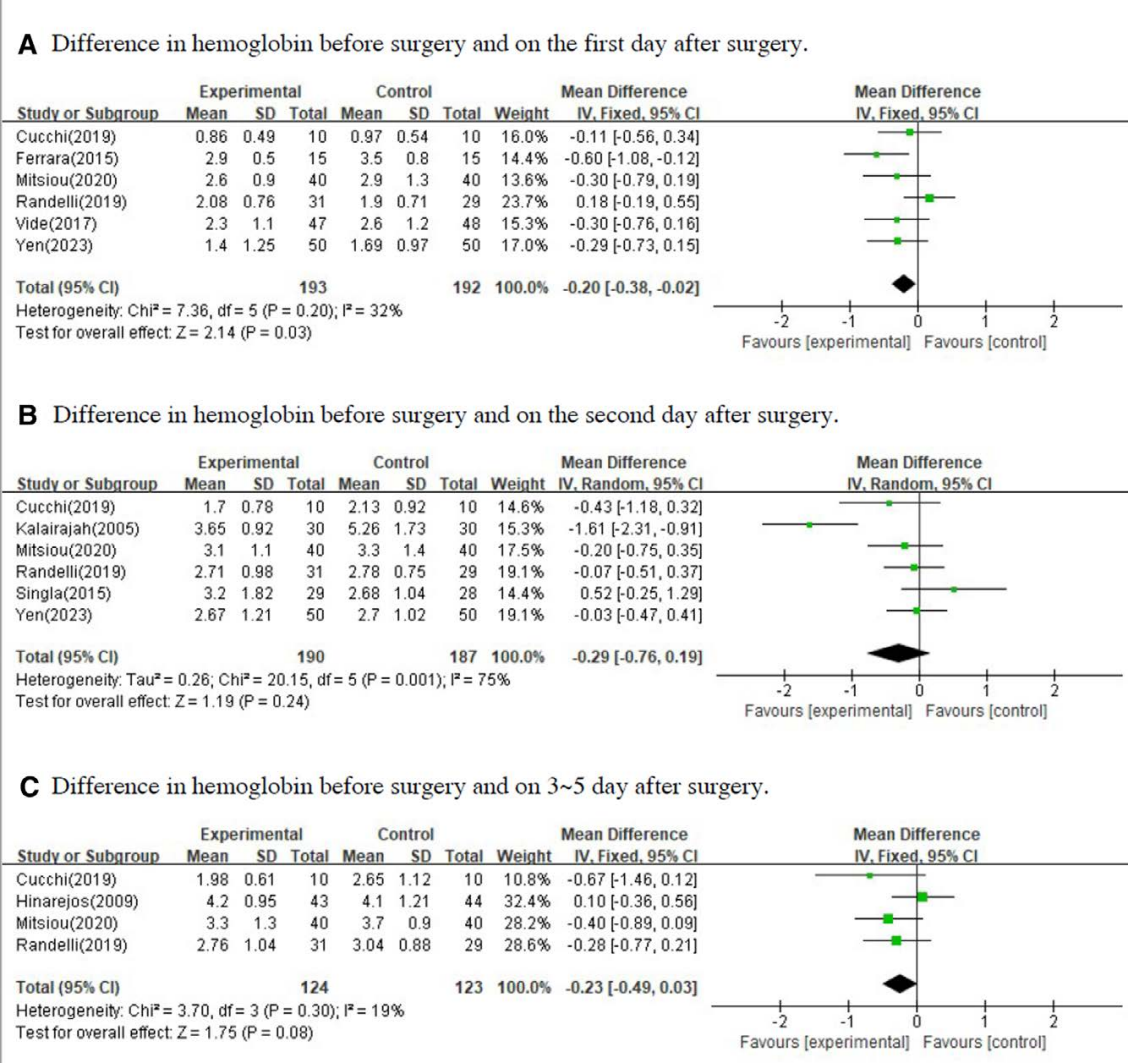
Forest plots for difference in hemoglobin was measured before surgery and after surgery between the group that underwent surgery by violating intramedullary cavity of the femur and the group that underwent surgery without violating intramedullary cavity of the femur. (A) Difference in hemoglobin before surgery and on the first day after surgery. (B) Difference in hemoglobin before surgery and on the second day after surgery. (C) Difference in hemoglobin before surgery and on 3 to 5 day after surgery.

**Figure 4.**
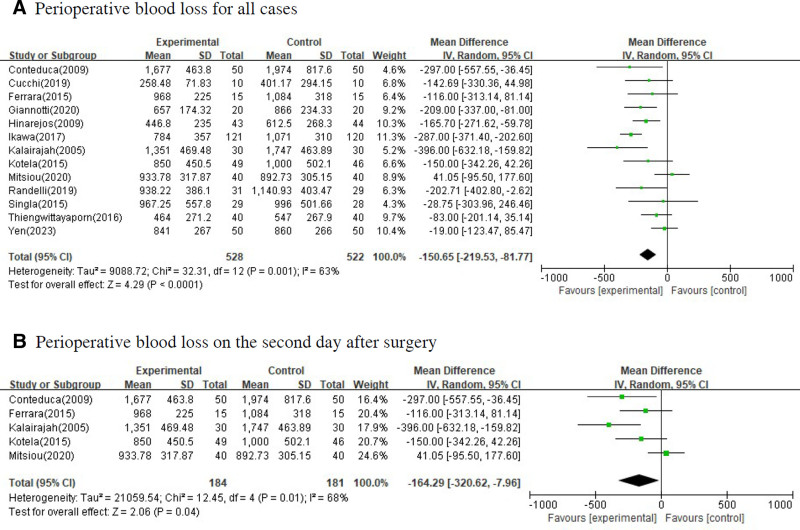
Forest plots for total blood loss between the group that underwent surgery by violating intramedullary cavity of the femur and the group that underwent surgery without violating intramedullary cavity of the femur. (A) Total blood loss for all cases; (B) total blood loss on the second day after surgery.

**Figure 5.**
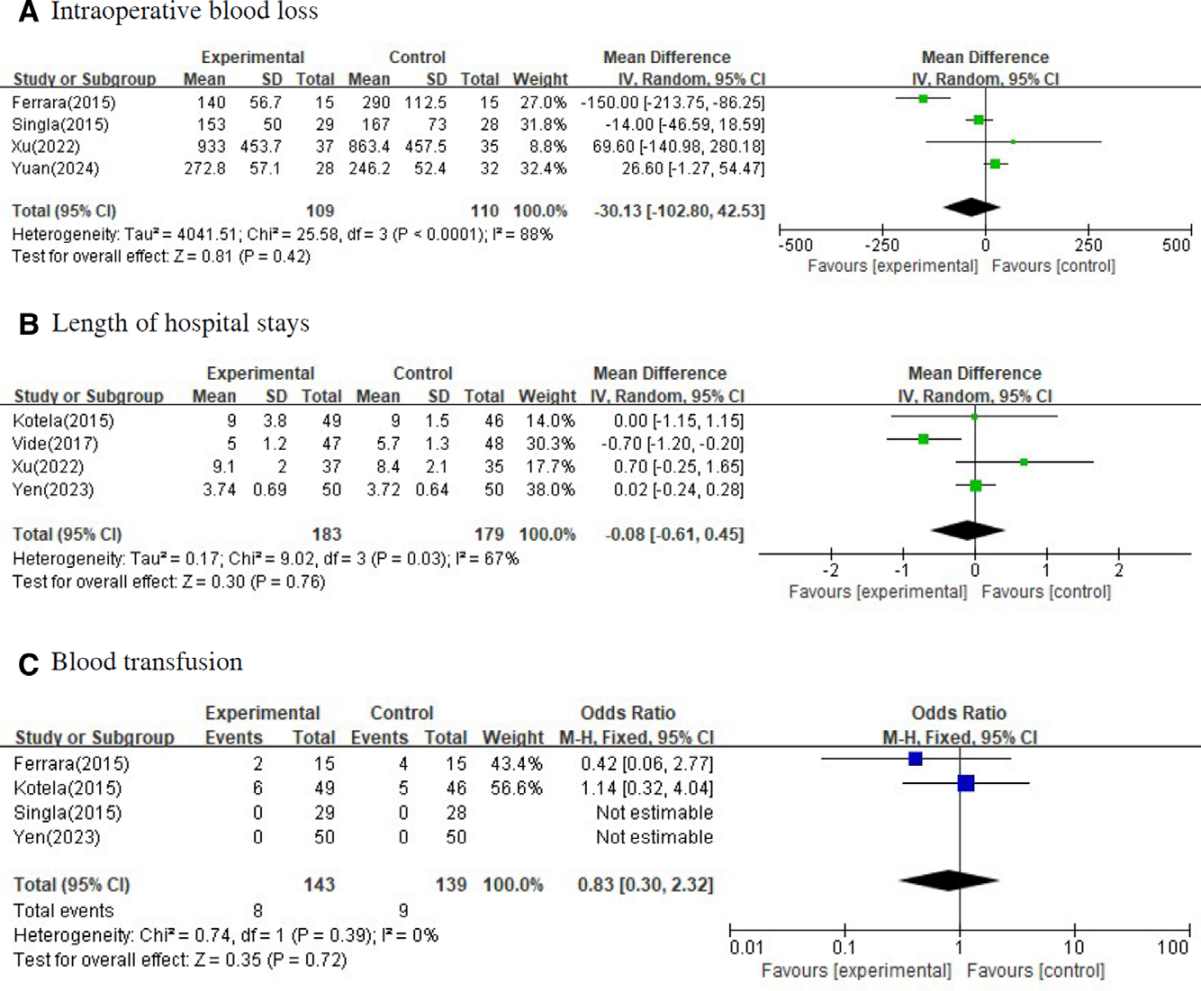
Forest plots for intraoperative blood loss, length of hospital stays, and blood transfusion between the group that underwent surgery by violating intramedullary cavity of the femur and the group that underwent surgery without violating intramedullary cavity of the femur.

In performing meta-analysis, a fixed effect model was used when the *P*-value was >.05 as a result of the homogeneity test, and a random effect model was used when the *P*-value was <.05. In the forest plot, mean difference and 95% confidence interval were presented for quantitative variables and odds ratio and 95% confidence interval were presented for qualitative variables. Figure 3 shows the difference in hemoglobin was measured before surgery and after surgery between the group that underwent conventional TKA and the group that underwent navigation or robot-assisted TKA or PSI. Figure 4 shows the perioperative blood loss for all cases and cases on the second day after surgery between 2 groups. Figure 5 shows the intraoperative blood loss, length of hospital stays and blood transfusion between 2 groups.

There was statistically significant difference in hemoglobin between the 2 groups before surgery and 1 day after surgery. The effect size of the difference in hemoglobin between before surgery and 1 day after surgery by 2 groups was −0.20 (95% CI: −0.38 to −0.02, *P*-value = .030). There was no statistically significant difference in hemoglobin between the 2 groups before surgery and 2 days after surgery and, before surgery and 3 to 5 days after surgery. The effect size of the difference in hemoglobin between before surgery and 2 days after surgery by 2 groups was −0.29 (95% CI: −0.76 to 0.19, *P*-value = .240). The effect size of the difference in hemoglobin between before surgery and 3 to 5 days after surgery by 2 groups was −0.23 (95% CI: −0.49 to 0.03, *P*-value = .080). Although there were not statistically significant on the 2nd day and 3rd to 5th day, the total effect size of difference in hemoglobin between 2 groups were −0.2 on the 1st day, −0.29 on the 2nd day and −0.23 on the 3rd to 5th day. Therefore, it can be seeming that the difference in hemoglobin before and after surgery in the group that underwent navigation or robot-assisted TKA or PSI significantly decreased less than the group that underwent conventional TKA.

There was statistically significant difference in perioperative blood loss. The effect size of the perioperative blood loss for all cases by 2 groups was −150.65 (95% CI: −219.53 to −81.77, *P*-value < .001). The effect size of the perioperative blood loss on the second day after surgery by 2 groups was −164.29 (95% CI: −320.62 to −7.96, *P*-value = .040). This means that the perioperative blood loss in the group that underwent navigation or robot-assisted TKA or PSI was significantly smaller than the group that underwent conventional TKA.

There was no statistically significant difference in intraoperative blood loss, length of hospital stays, and blood transfusion. The effect size of the intraoperative blood loss, length of hospital stays and blood transfusion by 2 groups were −30.13 (95% CI: −102.80 to 42.53, *P*-value = .420), −0.08 (95% CI: −0.61 to 0.45, *P*-value = .760), and 0.83 (95% CI: 0.30 to 2.32, *P*-value = .720), respectively.

### 3.4. Sensitivity analysis

The RMSE values for the hemoglobin change before surgery and 1 day after surgery, before surgery and 2 days after surgery, and before surgery and 3 to 5 days after surgery were calculated as 0.05, 0.11, and 0.07, respectively. The RMSE values for the perioperative blood loss for all cases and perioperative blood loss on the second day were calculated as 10.29 and 40.03, respectively. The RMSE values for the intraoperative blood loss, length of hospital stays, and blood transfusion were calculated as 23.03, 0.11, and 0.26, respectively. The RMSE value of each outcome was calculated to be <15% of the difference between the 95% lower limit and upper limit of the effect size. It can be seen that the overall effect size is not sensitive depending on the study.

### 3.5. Publication bias

On the basis of a few distinct methods, 2 of the authors (W.K.C. and S.G.K.) individually assessed the risk of bias. The risk of publication bias was determined using a funnel plot and the Egger test. A funnel plot was produced to investigate the risk of publication bias. Performing a visual inspection suggested some funnel plot asymmetry. In addition, the Egger test was not statistically significant in all cases, indicating that the observation of asymmetry was not supported and that there may not be a risk of publication bias.

## 4. Discussion

Navigation or robot-assisted TKA, as well as PSI, have been developed to attain optimal mechanical alignment of the femorotibial joint, which is crucial for ensuring longevity of the implant.^[9]^ All 3 surgical techniques, navigation or robot-assisted TKA, as well as PSI offer the incidental advantage of reduce bleeding within the femoral cavity as they do not require the use of an intramedullary guiding rod.^[10]^ Previous research has primarily focused on comparative analysis of each surgical technique and conventional TKA to determine their efficacy in achieving appropriate femorotibial alignment, with additional analysis on differences in perioperative blood loss and the frequency of blood transfusions.^[32]^ However, the results varied among different authors.^[11,12]^ Therefore, the authors grouped navigation or robot-assisted TKA, or PSI, which do not require invasion of the femoral cavity, into 1 category and systematically analyzed them with conventional TKA in terms of perioperative blood loss and transfusion frequency.

The analysis revealed the following findings.

Firstly, there was no statistically significant difference in intraoperative blood loss between the groups undergoing navigation or robot-assisted TKA, or PSI, and conventional TKA. However, in terms of perioperative blood loss, the group undergoing navigation or robot-assisted TKA, or PSI, showed a statistically significant lower amount compared to the conventional TKA group. In each RCT study, perioperative blood loss was measured using different methods. In Yen study,^[17]^ perioperative blood loss was estimated and analyzed by summing up both the expected blood loss based on the decrease in hemoglobin levels and the amount of blood transfused in cases where transfusion was received. Additionally, in Kotela study,^[21]^ perioperative blood loss was analyzed by assessing the amount of blood drained over a 2-day period. The blood loss during surgery arises from both actual bleeding occurring on the bone cutting surface and surrounding tissues due to surgical trauma, as well as bleeding within the femoral cavity. However, due to the use of torniquets as hemostatic agents in most studies, blood loss has been significantly reduced. In particular, after the insertion of the prosthetic joint replacement, the amount of bleeding occurring from the bone surface significantly decreases. However, since bleeding, including that from the femoral cavity, continues after surgery, the total perioperative bleeding, is believed to be lower in the navigation or robot-assisted TKA or PSI group. Secondly, when comparing the decrease in hemoglobin levels after surgery, the group undergoing navigation or robot-assisted TKA, or PSI, tended to have lower hemoglobin decrease values up to the 5th postoperative day, with statistically significant results observed on the 1st postoperative day. In previous studies, there has been extensive analysis of postoperative hemoglobin decrease. However, most studies did not specify when post-TKA hemoglobin tests were conducted. Typically, hemoglobin levels after TKA reach their lowest point around 3 to 5 days post-surgery and then gradually begin to recover.^[33]^ Therefore, it is important to specify which postoperative day’s hemoglobin value is being compared when analyzing hemoglobin levels. For this reason, in this study, the timing of hemoglobin measurement after surgery was divided into postoperative days 1, 2, and 3 to 5 for analysis. Thirdly, there was no statistically significant difference in the number of patients who received transfusion after surgery between the 2 groups. In most studies conducted, it is believed that even in cases of conventional surgery, the amount of perioperative bleeding is reduced through the use of torniquet as hemostatic agents, the administration of tranexamic acid, and surgical techniques such as plugging the femoral cavity with bone plugs.^[4]^

## 5. Conclusions

Navigation or robot-assisted TKA, or PSI, exhibited lower perioperative blood loss compared to conventional TKA. Additionally, they showed lower decreases in hemoglobin levels postoperatively. With these findings, it can be concluded that navigation or robot-assisted TKA, or PSI, may be considered as a selective option for reducing postoperative bleeding in TKA.

## Author contributions

**Conceptualization:** Sang Gyu Kwak, Hee Chan Kim, Jae Bum Kwon, Won-Kee Choi.

**Data curation:** Sang Gyu Kwak, Hee Chan Kim, Won-Kee Choi.

**Formal analysis:** Jae Bum Kwon.

**Validation:** Jae Bum Kwon.

**Visualization:** Sang Gyu Kwak, Jae Bum Kwon, Won-Kee Choi.

**Writing – original draft:** Sang Gyu Kwak, Won-Kee Choi.

**Writing – review & editing:** Hee Chan Kim, Won-Kee Choi.
